# An analysis of differential gene expression in peripheral nerve and muscle utilizing RNA sequencing after polyethylene glycol nerve fusion in a rat sciatic nerve injury model

**DOI:** 10.1371/journal.pone.0304773

**Published:** 2024-09-04

**Authors:** Samantha N. Weiss, Joseph M. Legato, Yichuan Liu, Courtney N. Vaccaro, Renata Pellegrino Da Silva, Sandra Miskiel, Grace V. Gilbert, Hakon Hakonarson, David A. Fuller, Russell J. Buono

**Affiliations:** 1 Department of Biomedical Sciences, Cooper Medical School of Rowan University, Camden, New Jersey, United States of America; 2 Bone and Joint Institute, Cooper University Hospital, Camden, New Jersey, United States of America; 3 Center for Applied Genomics, The Children’s Hospital of Philadelphia, Philadelphia, Pennsylvania, United States of America; Changchun Institute of Applied Chemistry, Chinese Academy of Sciences, CHINA

## Abstract

Application of polyethylene glycol (PEG) to a peripheral nerve injury at the time of primary neurorrhaphy is thought to prevent Wallerian degeneration via direct axolemma fusion. The molecular mechanisms of nerve fusion and recovery are unclear. Our study tested the hypothesis that PEG alters gene expression in neural and muscular environments as part of its restorative properties. Lewis rats underwent unilateral sciatic nerve transection with immediate primary repair. Subjects were randomly assigned to receive either PEG treatment or standard repair at the time of neurorrhaphy. Samples of sciatic nerve distal to the injury and tibialis muscle at the site of innervation were harvested at 24 hours and 4 weeks postoperatively. Total RNA sequencing and subsequent bioinformatics analyses were used to identify significant differences in differentially expressed genes (DEGs) and their related biological pathways (p<0.05) in PEG-treated subjects compared to non-PEG controls. No significant DEGs were identified in PEG-treated sciatic nerve compared to controls after 24 hours, but 1,480 DEGs were identified in PEG-treated tibialis compared to controls. At 4 weeks, 918 DEGs were identified in PEG-treated sciatic nerve, whereas only 3 DEGs remained in PEG-treated tibialis compared to controls. DEGs in sciatic were mostly upregulated (79%) and enriched in pathways present during nervous system development and growth, whereas DEGs in muscle were mostly downregulated (77%) and related to inflammation and tissue repair. Our findings indicate that PEG application during primary neurorrhaphy leads to significant differential gene regulation in the neural and muscular environment that is associated with improved functional recovery in animals treated with PEG compared to sham non-PEG controls. A detailed understanding of key molecules underlying PEG function in recovery after peripheral nerve repair may facilitate amplification of PEG effects through systemic or focal treatments at the time of neurotmesis.

## Introduction

Peripheral nerve injuries (PNIs) result in a continuum of symptoms ranging from mild discomfort to dramatic motor or sensory disturbances [1]. The majority of PNIs occur in the setting of trauma, specifically from motor vehicle accidents (46.4%), penetrating trauma (23.9%), and falls (10.9%) [2]. It has been posited that iatrogenic nerve injuries account for an additional 17.4% of PNIs [3]. Furthermore, studies have shown that up to 1.6% of all patients suffering traumatic upper- or lower-limb injuries were complicated by permanent, life-altering nervous system deficits [4]. Currently, primary neurorrhaphy is considered the gold standard of treatment for PNI which requires the anatomic re-approximation of severed nerve endings [5, 6]. Postoperative outcomes are dependent upon multiple variables, including the location of the PNI, patient age at time of PNI, and time from PNI to operative repair [7–9]. Despite medical advances, patient satisfaction regarding motor and sensory deficits is often low, with those suffering from complete transection or ablation type injuries reporting the lowest satisfaction [10–13].

Many clinicians attribute these suboptimal motor and sensory outcomes to Wallerian degeneration [14]. At the time of primary neurorrhaphy, the epineurium of the proximal and distal nerve segments is reapproximated, and the distal axonal segments are known to undergo Wallerian degeneration [6, 15]. Regeneration of the distal nerve segment then occurs at a rate of 1–3 mm/day, which facilitates re-innervation of downstream target tissues, albeit incompletely and nonspecifically [16, 17]. Phagocytes clear neuronal debris following Wallerian degeneration, while Schwann cells play an important supporting role during nerve regeneration by chemically stimulating neural growth [18–21]. In fact, the initial death of supporting Schwann cells following PNI is the principal limiting factor in nerve regeneration [22]. While the nerve may eventually grow to full length, downstream target muscles can become atrophic and fibrotic, thereby suffering irreparable damage in the interim [23].

Polyethylene glycol (PEG) is a synthetic, biodegradable material capable of fusing severed axolemmal membranes in PNIs [24]. A PEG protocol utilizing a series of solutions with varying tonicities and calcium concentrations was first developed and optimized by Bittner [25–31]. This protocol prevents Wallerian degeneration in the distal axonal segment in the setting of PEG-augmented primary neurorrhaphy [32]. Successful PEG fusion can be confirmed intra-operatively by assessing complex action potentials (CAPs) via demonstrating conduction across the coaptation site, as well as through complex motor action potentials (CMAPs) via demonstrating visible contraction of a downstream muscle target, traditionally the tibialis anterior muscle [33, 34]. This protocol has utility not only in immediate postoperative clinical outcomes in both animal and limited human studies, but also permits novice surgeons or those without substantial microsurgical training to achieve fusion rates of 95% or higher [35, 36]. However, the exact molecular mechanisms facilitating these benefits of PEG has not yet been elucidated.

Transcriptionally regulated genes have been identified at various timepoints following rat sciatic nerve injury, with maximal differential gene expression occurring 4 weeks post-repair [37]. Furthermore, whole transcriptome analyses of denervated rat gastrocnemius muscle demonstrated differential gene expression at various postoperative timepoints, suggesting certain RNA components may represent potential therapeutic targets to address irreparable muscle atrophy following PNI [38, 39]. In an effort to elucidate the molecular basis behind the non-rejection of living allogenic allografts in non-matched animals with no immunosuppression following PEG application, studies were performed using electron microscopy, RT-PCR, and immunohistochemistry and identified attenuation of inflammation and immune rejection markers in PEG treated tissues [39, 40]. Smith et al. are the first to investigate the coding transcriptome of PEG-fused rat sciatic nerve allografts using RNA sequencing in this model using outbred Sprague -Dawley rats. Molecular pathways associated with innate and adaptive immunity were demonstrated to be downregulated, whereas connective tissue development, extracellular matrix remodeling, and Schwann cell pathways were all upregulated [41].

The current study is the first to our knowledge to investigate global differential gene expression via RNA sequencing in both neural and muscle tissues following transection of rat sciatic nerve and PEG-augmented primary neurorrhaphy. Identification of key genetic and molecular pathways associated with the enhanced clinical outcomes following PEG-augmented primary neurorrhaphy may provide novel insight regarding potential molecular targets to reduce Wallerian degeneration.

## Materials and methods

### Ethics statement

This study was carried out in strict accordance with the recommendations in the Guide for the Care and Use of Laboratory Animals of the National Institutes of Health. The protocol was approved by the Institutional Animal Care and Use Committee of Cooper University Hospital (IACUC Protocol Number: 2019–004).

### Randomization of subjects

**Fig 1:**
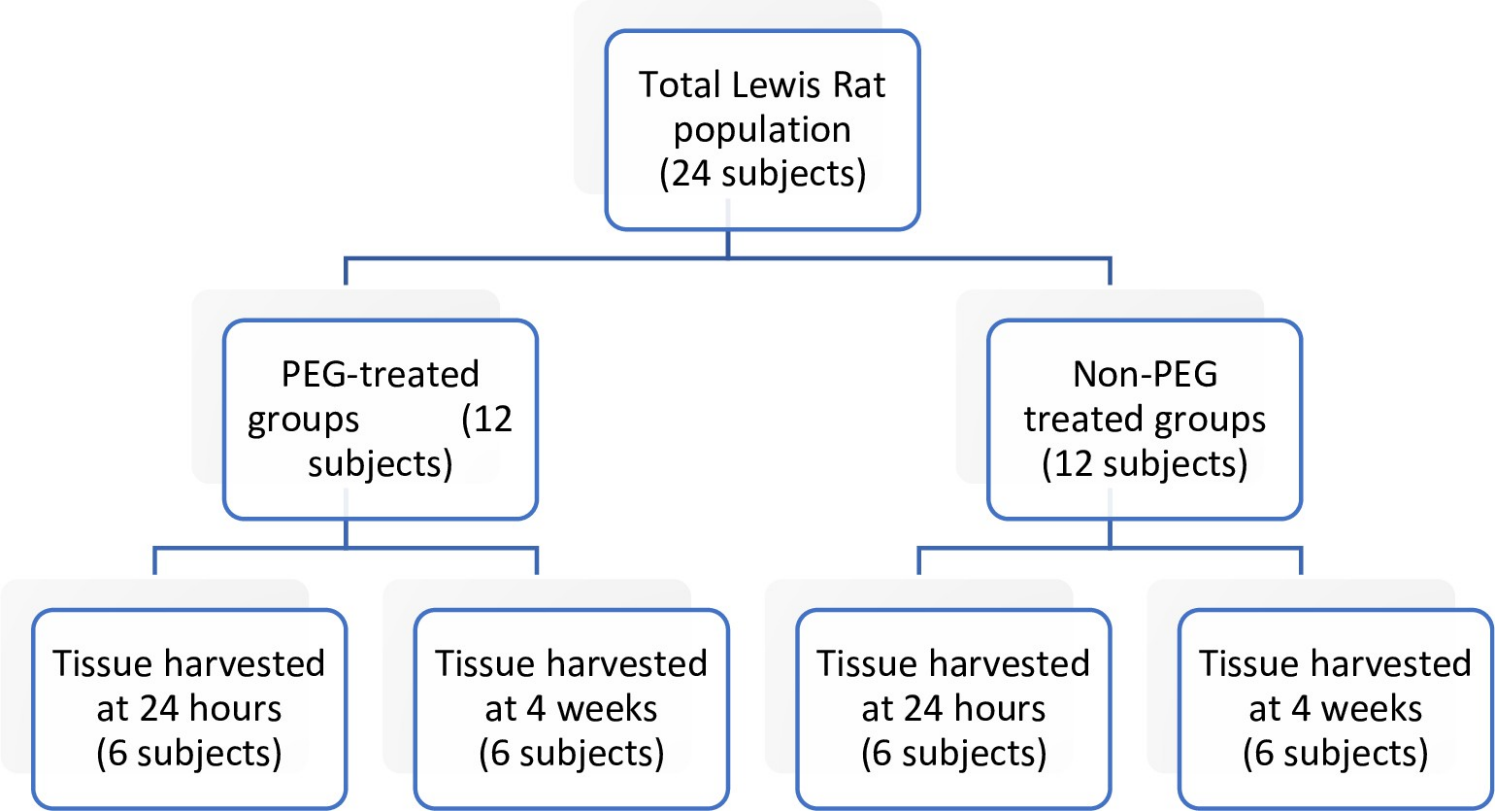
Experimental design demonstrating grouping of subjects.

Twenty-four male Lewis rats were randomly assigned to undergo primary neurorrhaphy with or without PEG fusion. Of the 12 subjects in each group, half (n = 6) were randomly assigned for tissue harvest at either 24 hours or 4 weeks postoperatively as shown in Fig 1.

Lewis rats are an inbred strain, meaning that consistent sibling mating has facilitated genetic homogeneity. Thus, the variability of genomic sequences between individual subjects is minimized, facilitating succinct differential gene expression data. Furthermore, Lewis rats have the added benefit of an apparent lower risk of potential autoamputation postoperatively, which has been documented in this and other rat species.

### Anesthesia

Thirty minutes prior to incision, subjects received one dose (1.2 mg/kg) of 72-hour sustained release buprenorphine (ZooPharm, Windsor, CO) and one dose (4 mg/kg) of 72-hour sustained release meloxicam (ZooPharm, Windsor, CO) subcutaneously for analgesia. Anesthesia was induced via isoflurane (4%) in oxygen delivered to a plastic induction chamber and was subsequently maintained with additional isoflurane (2%) delivered via nosecone intraoperatively. Depth of anesthesia was assessed prior to incision by lack of pedal reflex to toe pinch. Ophthalmic eye lubricant was administered to prevent dry eye discomfort during the procedure. Following anesthesia, the incision site was shaved, and underlying skin was scrubbed three times with alternating applications of betadine and isopropyl alcohol (70%) before being draped appropriately to ensure sterility. Normothermia was maintained via a heating pad with circulating warm water.

### Index surgery

Aseptic surgery was performed on three separate calendar days using standard microsurgical techniques under loupe magnification and microscope magnification (Leica WILD M690). The sciatic nerve was exposed through a three-centimeter posterolateral longitudinal straight skin incision from the hip to the knee joint. The trifurcation of the sciatic nerve into the tibial, peroneal, and sural branches was exposed through the interval between the gluteus superficialis and biceps femoris muscles. After confirming functionality of native sciatic nerve and downstream tibialis anterior muscle via observation of CMAPs, a transverse laceration located one centimeter proximal to the sciatic trifurcation above the popliteal fossa was created using an 11-blade scalpel over a wooden tongue depressor. Confirmation of complete transection was achieved via observation of separation of the 2 nerve endings following transection.

### Primary neurorrhaphy +/- peg fusion

A series of solutions originally defined by Bittner [25] and more recently elaborated upon by Ghergherehchi [31] was applied in succession to facilitate PEG fusion for all subjects randomly assigned to the experimental cohorts (N = 12). In brief, the nerve segments were irrigated with 0.5 mL of calcium-free, hypotonic solution (Plasma-Lyte A, Baxter Healthcare Corporation, Deerfield, IL). Then, 1–2 drops of 1% methylene blue antioxidant solution (Acros Organics, Geel, Belgium) were applied to both nerve ends for one minute. Next, the proximal and distal nerve segments were primarily repaired by experienced microsurgeons via placement of 9–0 nylon epineural micro-sutures. The coaptation site was then treated with 0.5 mL 50% PEG w/w solution in deionized water (Sigma Life Science, St. Louis, MO) for 90 seconds to facilitate axolemmal fusion. The PEG solution was finally irrigated away with Lactated Ringer’s solution (B. Braun Medical, Bethlehem, PA).

Axolemmal fusion was confirmed by observation of CMAPs as evidenced by visible contraction of the tibialis anterior muscle distal to the nerve coaptation site after PEG augmented neurorrhaphy as previously published by our group [42]. In brief, our method to confirm a successful PEG fusion was to stimulate the nerve proximal to the PEG fusion with a physiologic stimulation (0.1 mA) and observe for muscle activation distally (ankle plantar or dorsiflexion). The current at 0.1 mA was chosen based upon work with this model studying both PEG and non-PEG neurorrhaphy. In confirming our experimental setup, we found that a higher nerve stimulation could cause muscle activation distally even across a suture neurorrhaphy without a PEG fusion. We observed that 0.3 mA was a threshold for us above which a signal could potentially transmit even across standard suture neurorrhaphy. We confirmed also in the non-PEG neurorrhaphy legs that the distal muscles did not twitch with a stimulus of 0.1 mA intraoperatively after primary neurorrhaphy. Our prior publication documents this protocol and includes an image of the PEG fusion protocol [42].

For subjects randomly assigned to the control cohorts (N = 12), the PEG fusion protocol was not employed. Rather, the transected sciatic nerve was primarily repaired via placement of 9–0 nylon epineural micro-sutures only. All surgical wounds in all subjects regardless of assigned cohort were closed with surgical wound clips (Fine Science Tools, Foster City, CA). Following wound closure, 0.25% bupivacaine (8 mg/kg) was injected subcutaneously at the incision site for additional postoperative analgesia.

### Interim animal care and observation

All subjects were monitored for signs of distress following the index surgery. Specifically, the research and veterinary teams worked jointly providing post operative care and observing for any signs of distress. Daily monitoring and documentation for development of postoperative complications, including pain, infection, weight loss, or other disability, was shared amongst the laboratory and veterinary teams. In additional to the routine care, we observed the surgical legs for function thru observation gait evaluations. We evaluated for active ankle dorsiflexion in all the surgical legs. In the PEG neurorrhaphy legs, we confirmed active ankle dorsiflexion in all the surgical legs. In all the non-PEG neurorrhaphy legs, no active ankle dorsiflexion was observed throughout the postoperative period. Some heel and skin changes were noted in the non-PEG neurorrhaphy legs including clawing of the toes, ankle contractures and mild skin ulceration (heel pad changes) all consistent with denervation. No full thickness skin ulcers were observed.

### Secondary procedure with tissue harvest

All subjects underwent a second surgery to facilitate sciatic nerve and tibialis anterior sample collection at either 24 hours or 4 weeks following the initial index procedure. We decided on two time points to interrogate based on the work of Yi and colleagues [37] that followed the course of RNA expression using microarrays at multiple time point across 4 weeks after sciatic nerve transection. We chose an early timepoint of 24 hours and a late timepoint of 4 weeks to compare with the published work of this prior study. The same anesthesia protocol was utilized for this secondary surgery as was employed for the index surgery. Surgical access was achieved through the original incision site. The sciatic nerve was again exposed through the interval between the gluteus superficialis and biceps femoris musculature. Approximately 1 cm of sciatic nerve distal to the primary neurorrhaphy site and 50 mg of tibialis anterior muscle were harvested and stored in RNA Later Solution (Sigma-Aldrich, St. Louis, MO, USA) until all animal surgeries were complete to facilitate bulk processing of all samples. Subjects were euthanized via isoflurane overdose (>5%) with subsequent bilateral pneumothoraces.

### RNA extraction

Total RNA was extracted from samples by homogenizing tissues using the Maxwell® Kit on a Promega robotics platform according to the manufacturer’s instructions. The method used paramagnetic particles, which provide a mobile solid phase to optimize sample capture, washing, and subsequent purification of nucleic acid. Maxwell® Instruments (Promega, Madison, WI, USA) are magnetic particle-handling instruments that efficiently bind nucleic acids to the paramagnetic particle in the first well of a prefilled cartridge. RNA purity was measured by determination of the ratio for absorbance at 260 nm to absorbance at 280 nm (A260 nm/A280 nm) using a NanoDrop 8000 (Thermo, CA, USA). RNA integrity was also evaluated with the Agilent TapeStation System (Agilent, Santa Clara, CA, USA) determining RIN-values by gel electrophoresis. Approximately 50 mg w/w of tibialis anterior muscle was used for extraction, while the smaller sciatic nerve sample measured ~5 mg w/w. Thus, concentrations of total RNA in tibialis anterior muscle samples were high (>10ug/uL) compared to that of sciatic nerve samples (<10 ug/uL), necessitating two different methods for library preparation as described below.

### RNA Sequencing: High concentration RNA in tibialis anterior tissue

Preparation of ~50 mg tibialis anterior muscle samples for total RNA sequencing on the Illumina platform requires a series of handlings to remove ribosomal RNA (rRNA), fragment the RNA, convert the RNA to double-stranded complementary DNA (cDNA), and efficiently ligate appropriate indexed adapters to produce libraries with strand orientation information. Removal of rRNA was accomplished via biotinylated, target-specific oligos combined with Ribo-Zero rRNA removal beads (Illumina Inc., San Diego, CA, USA). The Ribo-Zero Human/Mouse/Rat kit depletes samples of cytoplasmic rRNA, while the Ribo-Zero Gold Human/Mouse/Rat kit depletes samples of both cytoplasmic and mitochondrial rRNA. Following purification, the RNA was fragmented into small pieces using divalent cations under elevated temperature. The cleaved RNA fragments were copied into first strand cDNA using reverse transcriptase and random primers, followed by second strand cDNA synthesis using DNA Polymerase I and RNase H. A single ’A’ base was then added to these cDNA fragments with subsequent ligation of the adapter. The products were purified and enriched via PCR to create the final cDNA library. Ribosomal RNA-depleted, strand-specific RNA libraries were generated with the TruSeq Total Ribo-Zero rRNA Removal (Illumina, San Diego, CA, USA). Each QCed library was sequenced on an Illumina Novaseq6000 (Illumina, Inc. San Diego, CA, USA) using V1.5 chemistry in paired-end mode with a read length of 2x100bp, generating approximately 40M reads per sample. All protocols were performed following the manufacturer’s instructions.

### RNA sequencing: Low concentration RNA in sciatic nerve tissue

Total RNA from each nerve sample was prepared for sequencing using the Takara SMARTer® Stranded Total RNA-Seq Kit v2—Pico Input Mammalian (Takara Bio USA, San Jose, CA, USA). Approximately 5 ng of RNA was used to prepare the RNA libraries. Five cycles of PCR were performed before rRNA depletion and fifteen cycles during the last library amplification. Following rRNA depletion, cDNA libraries were created using the Zapr enzyme, which specifically targets rRNA sequences. The libraries were sequenced on an Illumina Novaseq6000 (Illumina, Inc. San Diego, CA, USA) using V1.5 chemistry. Depths of 30–44 million paired-end 200 bp reads were generated for each sample.

### Bioinformatics analyses

Three tissue samples were used from separate animals per cohort (N = 3). FASTQ files were aligned to rat genome using Illumina DRAGEN RNA pipeline. Differential expression tests for genes were performed by DESeq2 package [43] with default parameters. Samples with/without treatments were pair-wisely compared for postoperative time points of 24 hours and 4 weeks, respectively. Differentially expressed genes (DEGs) were considered significant at corrected p-values lower than 0.05, using correction for multiple testing via the Benjamini method. The DAVID bioinformatics platform [44], Gene Ontology (GO), Functional Mapping and Annotation of Genome-Wide Association Studies (FUMA; PMID: 29184056), and the Kyoto Encyclopedia of Genes and Genomes (KEGG), was used for functional enrichment and pathway analyses of differentially expressed genes. Validation of DEGs was not performed. In well-designed RNA sequencing studies with 3 biological replicates, it has been shown that only ~1.8% of all identified DEGs are likely false positives and that the majority of these have low expression levels and less than 1.5 fold change [45]. Thus, a well-designed RNA sequencing study does not necessarily benefit from validation of the top significant DEGs based on log fold change or adjusted p-value via qRT-PCR or other methods and may in fact increase time and cost without adding value to the final analysis.

## Results

Our results demonstrate an inverse relationship in differential gene expression among sciatic nerve and tibialis anterior muscle at 24 hours and 4 weeks post-repair as shown in Table 1.

**Table 1:** PEG induced differential gene expression in sciatic nerve and tibialis muscle.

	Number of genes differentially expressed in Sciatic Nerve	Number of genes differentially expressed in Tibialis Muscle
24 Hours	0	1480
4 Weeks	918	3

At 24 hours post-repair, no genes were significantly differentially expressed within PEG-treated sciatic nerve compared to control, whereas 1,480 genes were differentially expressed within PEG-treated tibialis muscle compared to control. Conversely, at 4 weeks post-repair, 918 genes were differentially expressed within PEG-treated sciatic nerve compared to control, whereas only 3 genes (*Fosb*, *Orai1*, *Rps15al3*) continued to be differentially expressed within PEG-treated tibialis muscle compared to control. The differentially expressed genes (DEGs) identified are both upregulated and downregulated as indicated by their direction of fold change comparing PEG to non-PEG treated. We focused on DEGs with significant adjusted p-values (< 0.05) and with >1.5-fold change in expression as potentially being the most biologically significant. We show that in tibialis muscle at 24 hours 77% (1143/1480) of the significant DEGs are downregulated and only 6% (93/1480) are upregulated by more than 1.5-fold. In contrast, we show in sciatic nerve at the 4-week time point that 64% (586/918) of the significant DEGs are upregulated and 5% (47/918) are downregulated by more than 1.5-fold. A complete list of the DEGs from the output of the DESeq2 analysis as described above is available in S1–S4 Tables (S1–S4 Tables).

**Fig 2:**
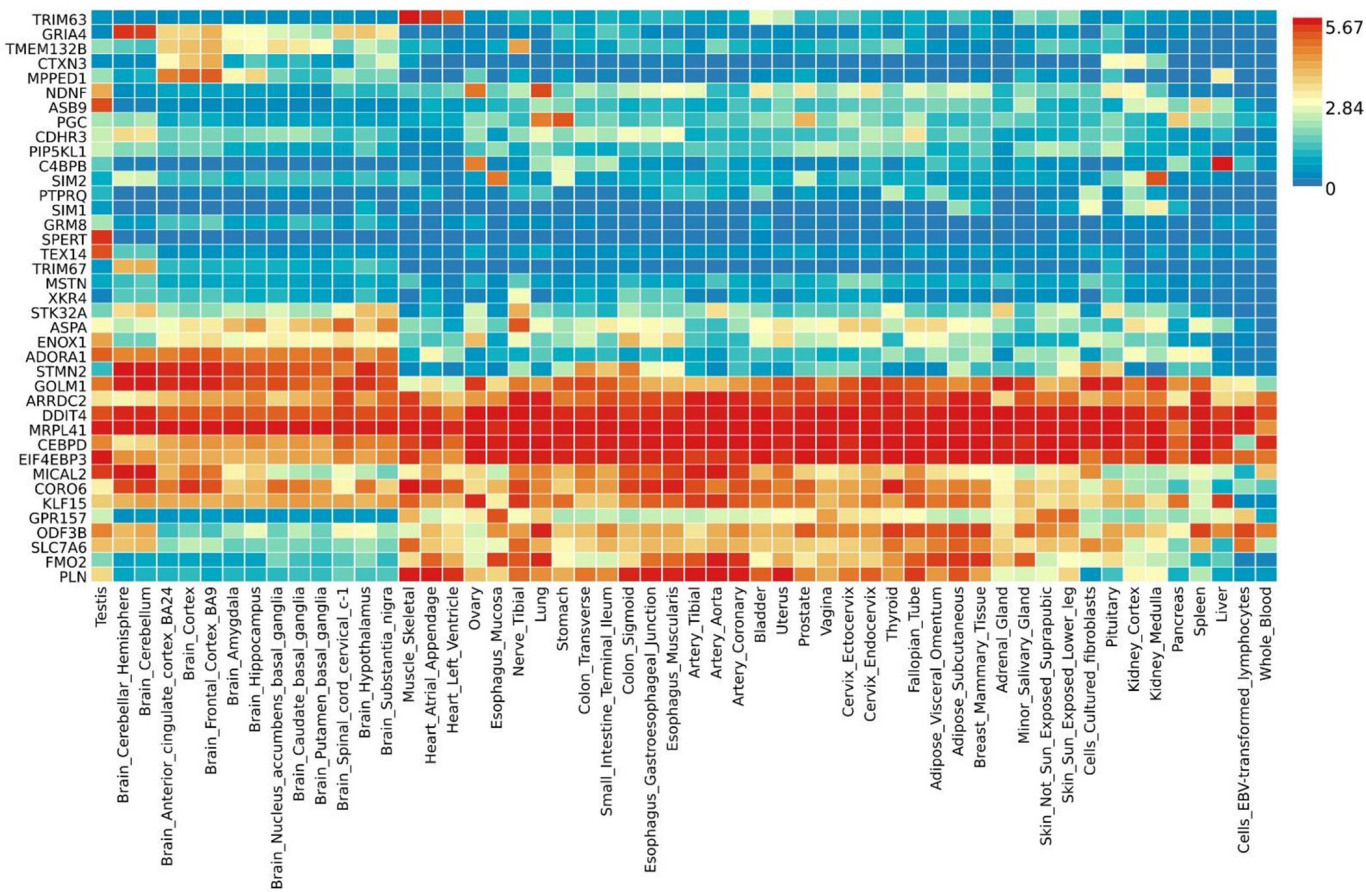
Heatmap of rat tibialis muscle DEGs upregulated by 1.5-fold or more at 24 hours after PEG treatment. Shows individual genes and their intensity of expression across 54 adult human tissues on the GTEX version 8 database. Red indicates high expression and blue indicates low expression for any given gene in different tissues. Intensity measures are only valid in the horizontal direction for a single gene across different tissues and are not valid vertically such that expression of many genes cannot be compared in one tissue.

We used the Gene2Function analysis in FUMA to input the 1480 and 918 DEGs identified in PEG treated tibialis muscle at 24 hours and sciatic nerve at 4 weeks respectively and compare them against the human GTEX database. We used all genes as background for our analysis. FUMA identified the known human homologs from our list of rat DEGs, and then compared our gene sets to the GTEX human tissue gene expression database. Not all loci identified in the Rat Genome Database used as reference for RNA sequencing can be identified by FUMA as it uses HUGO gene names or Entrez ID. In our data sets, 522/918 (57%) rat genome DEGs in sciatic nerve and 1182/1480 (80%) rat genome DEGs in tibialis muscle were recognized and matched to human genes by the algorithm. Indeed, most all the rat DEGs not recognized as human genes by the FUMA algorithm are yet uncharacterized genes encoding proteins of unknown function. GTEX represents gene expression data taken from 54 different human tissues from nearly 1000 adult individuals. Input of the DEGs as a gene name list generates output as a heatmap showing how the input DEGs are expressed across multiple adult human tissues. Summary statistics across DEG sets yield bar graphs that show significant upregulation and downregulation patterns across different adult tissues. Fig 2. Shows the heatmap after input of 93 DEGs upregulated by more than 1.5-fold in tibialis muscle 24 hours after nerve damage and PEG repair treatment. The heatmap contains 40 of the 93 DEGs due to discrepancies between rat genome loci nomenclature and the human loci in the GTEX database.

**Fig 3:**
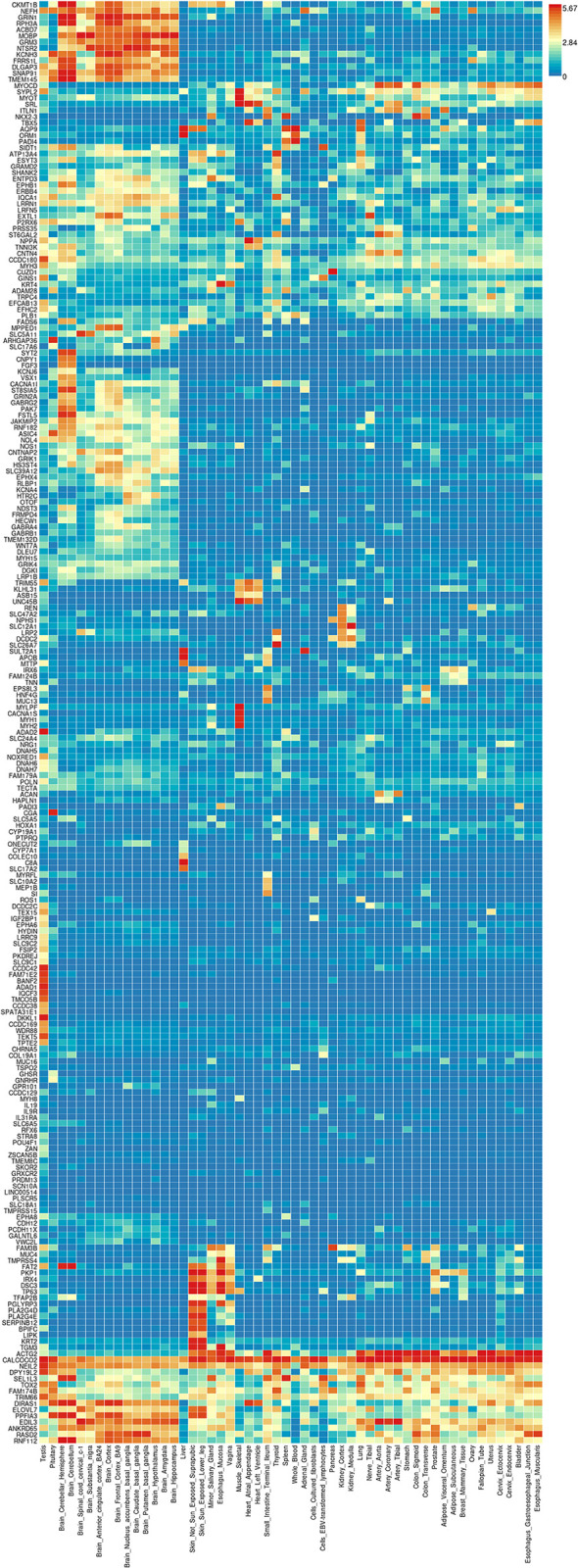
Heatmap of rat sciatic nerve DEGs upregulated by 1.5-fold or more at 4-weeks after PEG treatment. Shows individual genes and their intensity of expression across 54 adult human tissues on the GTEX version 8 database. Red indicates high expression and blue indicates low expression for any given gene in different tissues. Intensity measures are only valid in the horizontal direction for a single gene across different tissues and are not valid vertically such that expression of many genes cannot be compared in one tissue.

Note the DEG from our data set in row one of Fig 2. TRIM63, is highly expressed in skeletal and some cardiac muscle, but not in other tissues. The gene encodes a muscle specific protein involved in protein degradation and is highly expressed in skeletal muscle and cardiac muscle during atrophy. This finding is consistent with upregulation of this DEG in our data set and the muscle response to initial damage from denervation and exemplifies some of the gene level findings that can be made using FUMA analysis. The number one significant gene by adjusted p-value that was >1.5 log fold change in tibialis muscle at 24 hours was RGD1562029, a gene (KIAA2012 human homolog) encoding a protein of unknown function that may interact with gene products of the proteosome complex based on the STRING program on GeneCards. All DEGs for each analysis time point can be found in S1–S4 Tables (S1–S4 Tables). Fig 3. Shows the heatmap after input of 586 DEGs upregulated by more than 1.5-fold in sciatic nerve 4 weeks after nerve damage and PEG repair treatment. The heatmap contains 333 of the 586 DEGs due to discrepancies between rat genome loci nomenclature and the human loci in the GTEX database.

Fig 3 shows clustering of upregulated DEGs identified in sciatic nerve compared with general expression across 54 human tissues in the GTEX database including skeletal muscle and tibial nerve. Central nervous system tissues show increased clusters of high expression in both the upper and lower left corners of the heatmap relative to upregulated DEGs found in sciatic nerve at the 4-week time point. Two DEGs are highly expressed in all tissues suggesting constitutive expression for housekeeping functions; CALCOC2 is involved in actin cytoskeletal organization and as part of the immune system recognition of pathogens and NEIL1 which is involved in DNA damage repair. Of note, the input upregulated DEGs from sciatic nerve at the 4-week timepoint overlap with high expression skeletal muscle genes related to myosin filaments and components of the sarcoplasmic reticulum and T-tubule system (MYH1, MYH2, MYLPF, MYOT, SRL, SYPL2).

**Fig 4:**
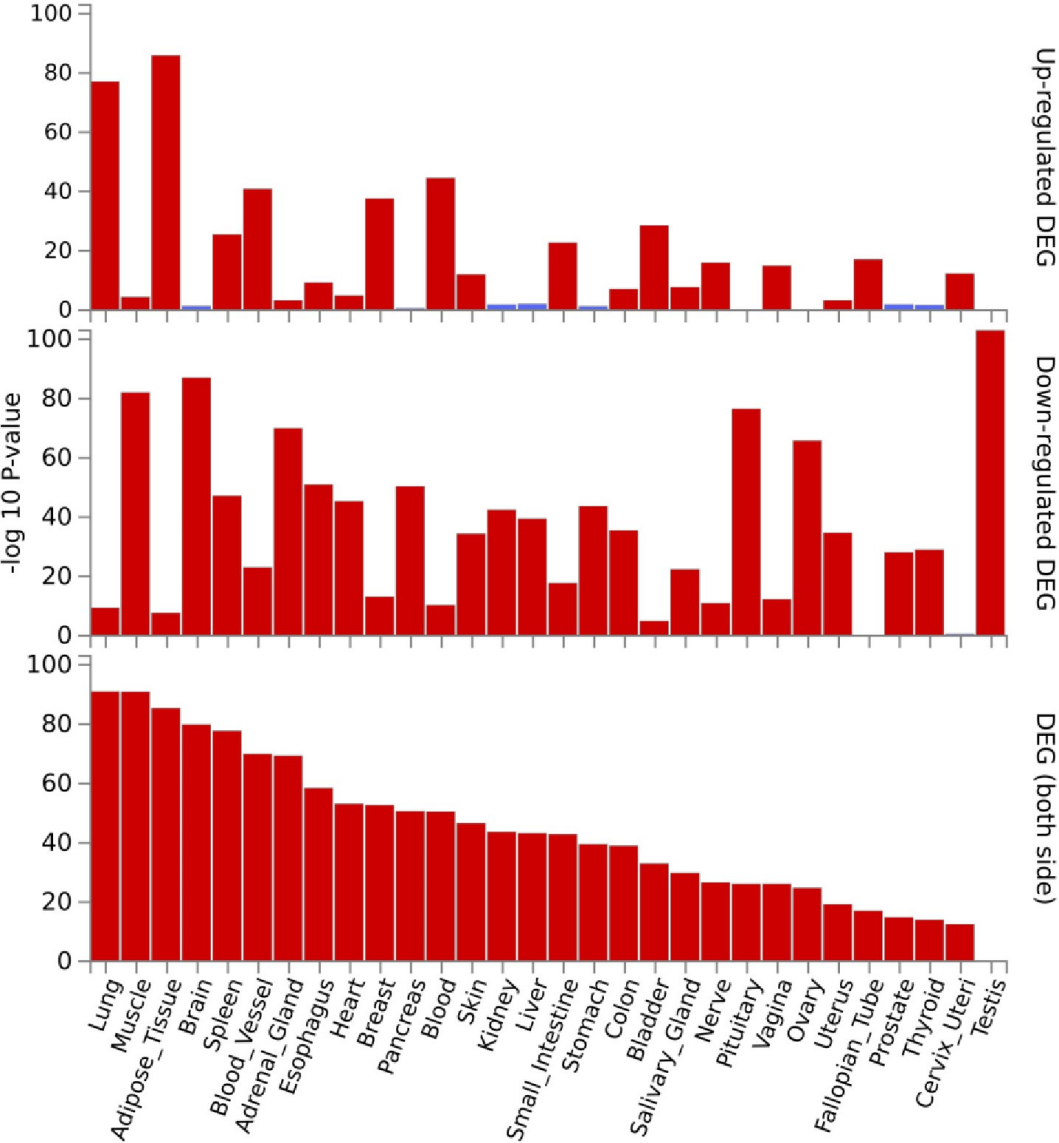
FUMA analysis of 1480 DEGs identified in tibialis muscle at 24 hours after PEG treatment and their qualitative expression in 30 different tissues in GTEX V8. Red bars indicate where input DEG gene sets show significant expression and overlap with GTEX V8 RNAseq data collected from 30 different tissues. Blue (and no) bars are not significant. The FUMA output shows upregulation and downregulation of all genes comparing each genes expression against all other genes and using t-statistics from RNA seq data analysis to determine direction of regulation.

Fig 4. Shows the FUMA analysis of summary statistics for the DEGs analyzed in the tibialis muscle at 24 hours and their qualitative expression in 30 different tissues.

Note in Fig 4. the second lane representing the overlap between the input DEGs from tibialis muscle at 24 hours and the skeletal muscle samples in the GTEX V8 database in all three panels. There is a high percentage overlap between the input DEGs and GTEX and most genes appear to be downregulated concurrent with our experimental findings. While these gene level analyses are possible, identifying individual DEGs of importance remains a challenge for RNA-sequencing studies. Gene Ontology (GO) and other web-based applications allow for the input of DEG sets from RNA sequence data and identifies how many DEGs are found in specific biological pathways and or cellular locations. The algorithms compare the number of genes in a DEG set that overlap with a set of validated genes connected by biological structure and function. The programs then generate p-values based on the number of DEGs observed versus the number of DGEs expected and includes correction for multiple testing. The GO platform can elucidate connections between genes at the molecular, biological process, cellular, tissue, and organismal levels.

While no differential genetic pathway regulation was identified in the sciatic nerve at 24 hours post-repair, there were numerous statistically significant genetic pathway regulations within sciatic nerve tissue at 4 weeks post-repair across multiple levels. Specifically, the GO functional enrichment analysis of biological processes identified 97 differentially regulated biological processes many of which are directly related to neural development, such as development of the nervous system, lipid metabolism and ion transport. The top 20 GO biological pathways based on p-value are shown in Table 2. The complete table is shown in S5 Table (S5 Table).

**Table 2:** Top 20 pathways (of 97 total) significantly affected by PEG in sciatic nerve at 4 weeks (GO Biological Process).

Term	P value	Corrected p value
Single-Multicellular Organism Process	2.92E-13	9.11E-10
System development	3.39E-13	9.11E-10
Multicellular Organism Development	5.47E-12	9.79E-09
Single-Organism Developmental Process	1.97E-10	2.52E-07
Animal Organ Development	2.35E-10	2.52E-07
Developmental Process	4.08E-10	3.65E-07
Anatomical Structure Development	4.83E-10	3.71E-07
Single-Organism Process	1.33E-09	8.92E-07
Nervous System Development	9.06E-08	5.40E-05
Ion Transport	2.61E-07	1.40E-04
Response to Organic Cyclic Compound	3.32E-07	1.62E-04
Cellular Response to Organic Substance	7.27E-07	3.17E-04
Response to Organic Substance	7.67E-07	3.17E-04
Tissue Development	9.45E-07	3.62E-04
Peripheral Nervous System Development	1.66E-06	5.57E-04
Cell Differentiation	1.66E-06	5.57E-04
Lipid Metabolic Process	2.56E-06	8.09E-04
Response to Endogenous Stimulus	4.47E-06	1.33E-03
Ion Transmembrane Transport	5.70E-06	1.61E-03
Sodium Ion Transport	1.06E-05	2.82E-03

The GO Cell Complement functional analysis found ten significant cellular pathway associations as a result of PEG application intraoperatively as shown in Table 3. These pathways include extracellular matrix production, plasma membrane repair, and generation of synapses including dendritic spines and terminal boutons (Table 3).

**Table 3:** Pathways significantly affected by PEG in sciatic nerve at 4 weeks (GO Cell Complement).

Term	P value	Corrected p value
Proteinaceous Extracellular Matrix	4.50E-08	1.53E-05
Extracellular Space	2.20E-05	3.73E-03
Terminal Bouton	6.16E-05	6.99E-03
Dendritic Spine	1.43E-04	1.22E-02
Integral Component of Plasma Membrane	2.17E-04	1.47E-02
Synapse	5.65E-04	3.01E-02
MHC Class II Protein Complex	6.20E-04	3.01E-02
Myosin Complex	7.55E-04	3.21E-02
Postsynaptic Membrane	8.74E-04	3.30E-02
Brush Border Membrane	1.34E-03	4.56E-02

Kyoto Encyclopedia of Genes and Genome (KEGG) analyses identified two additional pathways related to steroid synthesis that were significantly affected by PEG treatment in sciatic nerve at 4-weeks post-repair and these are shown in Table 4.

**Table 4:** Pathways significantly affected by PEG in sciatic nerve at 4 weeks (KEGG).

Term	P value	Corrected p value
Terpenoid Backbone Biosynthesis	3.21E-05	7.03E-03
Steroid Biosynthesis	1.89E-04	2.07E-02

Regarding tibialis anterior muscle, differential gene expression was most prominent at the 24-hour time point with 1,480 genes being identified as affected by PEG treatment. GO and KEGG functional enrichment analyses were again utilized to elucidate the specific genetic pathways comprised of the individual genes affected. GO Biological Processes identified 52 differentially regulated biological process pathways following intraoperative PEG application, with many inflammatory processes inherent to early tissue repair being prominent. The top 20 pathways based on p-value are shown in Table 5. The complete table is shown in S6 Table (S6 Table).

**Table 5:** Top 20 pathways (of 52 total) significantly affected by PEG in tibialis anterior muscle 24 hours (GO Biological Process).

Term	P value	Corrected p value
Cell Adhesion	4.61E-11	1.19E-07
Response to Lipopolysaccharide	5.49E-11	1.19E-07
Inflammatory Response	1.53E-10	2.21E-07
Cytokine-Mediated Signaling Pathway	3.64E-09	3.95E-06
Integrin-Mediated Signaling Pathway	4.65E-09	4.03E-06
Angiogenesis	2.73E-08	1.98E-05
Neutrophil chemotaxis	9.11E-08	5.65E-05
Positive Regulation of T Cell Proliferation	1.30E-07	6.44E-05
Positive Regulation of Cell Migration	1.34E-07	6.44E-05
Platelet Aggregation	1.49E-07	6.47E-05
Oxygen Transport	5.72E-07	2.26E-04
Collagen Fibril Organization	7.98E-07	2.89E-04
Cellular Response to Platelet-Derived Growth Factor Stimulus	9.73E-07	3.14E-04
Receptor-Mediated Endocytosis	1.01E-06	3.14E-04
Positive Regulation of Neutrophil Chemotaxis	1.72E-06	4.67E-04
Leukocyte Cell-Cell Adhesion	1.72E-06	4.67E-04
Innate Immune Response	2.46E-06	6.27E-04
Extracellular Matrix Organization	3.43E-06	8.27E-04
Response to Cytokine	4.77E-06	1.09E-03
Cartilage Development	5.41E-06	1.17E-03

It is interesting to note that the majority of the 1480 DEGs identified are downregulated, suggesting that PEG initially reduces the pro-inflammatory pathways induced by denervation injury.

GO Molecular Function identified an additional 28 pathways that are significantly affected by PEG treatment in muscle at the 24-hour time point, also comprised of pathways inherent to immediate attempts by damaged muscle to repair and regenerate tissue and extracellular matrix connections. These pathways are shown in Table 6.

**Table 6:** Pathways significantly affected by PEG in tibialis anterior muscle 24 Hours (GO Molecular Function).

Term	P value	Corrected p value
Calcium Ion Binding	7.13E-12	5.51E-09
Extracellular Matrix Binding	9.36E-12	5.51E-09
Collagen Binding	7.70E-10	3.02E-07
Heparin Binding	2.46E-09	7.25E-07
Protein Binding	2.28E-08	5.36E-06
Actin Filament Binding	4.83E-08	9.47E-06
Identical Protein Binding	8.53E-08	1.28E-05
Extracellular Matrix Structural Constituent	8.69E-08	1.28E-05
Scavenger Receptor Activity	1.65E-07	2.15E-05
Integrin Binding	2.62E-07	3.08E-05
Cell Adhesion Molecule Binding	3.39E-07	3.63E-05
Oxygen Transporter Activity	4.98E-07	4.88E-05
Fibronectin Binding	1.23E-06	1.11E-04
Actin Binding	1.37E-06	1.16E-04
Protein Homodimerization Activity	3.00E-06	2.35E-04
WW Domain Binding	1.94E-05	1.43E-03
Oxygen Binding	7.28E-05	5.04E-03
S100 Protein Binding	7.84E-05	5.13E-03
Carbohydrate Binding	1.10E-04	6.79E-03
Peroxidase Activity	1.59E-04	8.99E-03
Phospholipid Binding	1.68E-04	8.99E-03
Cadherin Binding Involved in Cell-Cell Adhesion	1.68E-04	8.99E-03
Laminin Binding	2.68E-04	1.37E-02
Glycoprotein Binding	3.34E-04	1.64E-02
Sialic Acid Binding	4.98E-04	2.35E-02
Cytokine Receptor Activity	5.73E-04	2.59E-02
Phosphatidylinositol Binding	6.50E-04	2.83E-02
GTPase Activity	8.72E-04	3.67E-02

KEGG analyses revealed an additional 27 pathways that are significantly altered by PEG treatment in muscle at this time point including common pathways regulating development and differentiation process, such as PI3K-Akt and Jak-STAT signaling pathways. Other regulatory signaling pathways are found as well as osteoclast differentiation and coagulation, complement and other immune system components. These pathways are shown in Table 7.

**Table 7:** Pathways significantly affected by PEG in tibialis anterior muscle 24 hours (KEGG).

Term	P value	Corrected p value
ECM-Receptor Interaction	3.21E-10	4.30E-08
Platelet Activation	1.83E-08	1.63E-06
Focal Adhesion	8.92E-08	5.97E-06
Cytokine-Cytokine Receptor Interaction	1.98E-07	1.06E-05
Hematopoietic Cell Lineage	9.35E-07	4.18E-05
Osteoclast Differentiation	4.18E-06	1.60E-04
Regulation of Actin Cytoskeleton	1.22E-05	4.10E-04
Natural Killer Cell Mediated Cytotoxicity	1.73E-05	5.14E-04
Leukocyte Transendothelial Migration	2.58E-05	6.93E-04
African Trypanosomiasis	4.98E-05	1.21E-03
PI3K-Akt Signaling Pathway	8.62E-05	1.93E-03
Proteoglycans in Cancer	1.00E-04	2.06E-03
Phagosome	1.15E-04	2.21E-03
Rap1 Signaling Pathway	1.61E-04	2.88E-03
Pertussis	1.78E-04	2.98E-03
Arrhythmogenic Right Ventricular Cardiomyopathy (ARVC)	2.42E-04	3.81E-03
Fc gamma R-Mediated Phagocytosis	3.18E-04	4.74E-03
Staphylococcus Aureus Infection	3.66E-04	4.98E-03
Chemokine Signaling Pathway	3.71E-04	4.98E-03
B Cell Receptor Signaling Pathway	1.13E-03	1.37E-02
Fc epsilon RI Signaling Pathway	1.13E-03	1.37E-02
Complement and Coagulation Cascades	1.70E-03	1.98E-02
Adipocytokine Signaling Pathway	2.48E-03	2.69E-02
Dilated Cardiomyopathy	2.52E-03	2.69E-02
Amoebiasis	2.61E-03	2.69E-02
Jak-STAT Signaling Pathway	3.62E-03	3.59E-02
Protein Digestion and Absorption	4.35E-03	4.16E-02

## Discussion

The use of PEG in repair of transected peripheral nerves is a promising development for the treatment of high-grade PNIs. A recent systematic review highlighted sixteen animal studies that all document superiority in outcomes following treatment with PEG via histological, electrophysiological, and/or behavioral measures [35]. However, the molecular basis facilitating these improved outcomes associated with intraoperative PEG fusion during primary neurorrhaphy is not clearly understood. To our knowledge, this is the first study utilizing RNA sequencing to investigate differential gene expression following PEG-augmented primary neurorrhaphy in both nerve and muscle tissues in a rat sciatic nerve transection model.

Prior work in a rat model investigating differential gene expression after nerve transection via mRNA microarray analysis was published by Yi et al [37]. Multiple time points were explored to interrogate gene expression in the distal degenerating nerve segment, including 0.5-, 1-, 6-, 12-, and 24-hours, 4 days, and 1-, 2-, 3-, and 4-weeks following nerve injury. Notably, Yi et al. demonstrated rapid Wallerian degeneration with minimal accompanying differential gene expression at early time points. Differential gene expression was reported to increase over time, ultimately peaking 4 weeks after initial injury with ~300 genes being affected overall. We decided on two time points to interrogate based on the work of Yi, an early timepoint of 24 hours and a late timepoint of 4 weeks. Our data are consistent in that our PEG treated sciatic nerve had minimal differential gene regulation at 24 hours, with maximal differential gene expression occurring 4 weeks postoperatively with 918 genes identified. Thus, when comparing our data to the results of Yi et al., intraoperative PEG application during primary neurorrhaphy increases the number of differentially regulated genes in rat sciatic nerve 3-fold at the 4 week time point. These genetic changes induced by PEG application likely contribute to the improved functional outcomes observed in PEG treated subjects compared to control and are likely associated with the postulated inhibition of Wallerian degeneration. Systematic screening of these genes for transcription factors and other potential “key regulatory” molecules is underway. The lack of significant DEG in sciatic nerve at 24 hours post-surgery is not clearly understood. The traumatized tissue appears to be rendered unresponsive initially and requires more than 24 hours to recover transcriptional function.

Weng et al. implemented RNA sequencing in a mouse model of sciatic nerve transection but focused on differential gene expression in the target muscle [38]. In this study, the transcriptome of atrophying gastrocnemius muscle was investigated at various timepoints following sciatic nerve injury, as opposed to the transcriptome of the injured sciatic nerve itself. Weng et al. report a similar trend to our data in that the number of differentially expressed noncoding RNAs in muscle tissues are highest immediately following PNI and subsequently decrease thereafter. However, in contrast to our study, they report relatively stable expression of 1,500 or more mRNAs in non-PEG treated mouse gastrocnemius muscle over the 4-week period. Our data demonstrate 1,480 genes differentially expressed at the 24-hour timepoint, with this number reducing to only three genes differentially expressed by 4 weeks in the PEG treated rat tibialis muscle. Combined with the fact that the majority of DEGs identified in tibialis muscle at 24 hours are downregulated and that only 3 DEGs remain at 4 weeks, these data suggest that PEG treatment inhibits mRNA expression in tibialis muscle over time. It is noteworthy that significant overlap exists in the KEGG pathway analyses between our results in muscle at 24 hours post nerve injury and the one-week timepoint discussed by Weng et al., including differential regulation of platelet activation, osteoclast differentiation, FC gamma R mediated phagocytosis, and other key pathways.

Of interest is that *FOSB* is one of the three genes differentially regulated in muscle at the 4-week time point. *FOSB* represents a potential “key regulatory” molecule as it encodes a protein that is a component of a transcription factor complex which controls expression of other genes. *FOSB* is part of a family of *FOS* genes that encode leucine zipper proteins, which dimerize with *JUN* gene family proteins. Collectively, these dimers form the transcription factor complex AP-1, which is used to induce and repress many other genes with functions related to cell proliferation, differentiation, and transformation. Given that *FOSB* expression at the 4-week time point in muscle may play a role in maintenance of inhibited Wallerian degeneration, this potential protective mechanism warrants further study. The other two genes encode a protein related to calcium homeostasis in muscle and heart and a subunit of the 40S ribosomal RNA complex (*Orai1*, *Rps15al3)*.

It is possible that other “key regulatory” molecules, such as transcription factors or tyrosine kinases, may be identified in subsequent gene level data analyses in the PEG treated tissues. These molecules have been well characterized as playing critical roles in differential gene expression related to cellular proliferation and differentiation. On inspection of data at the individual gene level generated from muscle tissue at the 24-hour time point, 77% of the gene expression changes are in the form of repression. However, in the gene level data from the nerve tissue at 4 weeks, over 79% of the gene expression pattern is upregulation. Identification of key regulatory molecules from gene level data related to these patterns is a challenge not yet discernable by bioinformatics programs and thus requires individual curation of each gene to search for functional relationships beyond what pathway analyses can accomplish. It is possible the gene expression differences are related to chromatin structure changes and epigenetic modification of DNA and histone proteins. In the future, these experiments could be repeated using ATAC or HiC sequencing to determine the role of chromatin modifications on differential gene expression in this system.

## Conclusion

Our data suggest that PEG-induced differential gene regulation in both distal nerve and muscle contributes to the inhibition of Wallerian degeneration and functional preservation in this rat sciatic nerve transection model. DEGs related to promoter function and mRNA processing could be viable targets for therapeutic intervention in the future. These new data document pathways that are differentially regulated in PEG compared to non-PEG treated nerve and muscle and open a window on the molecular landscape that could be used to develop specific treatment strategies for PNIs by augmenting and improving on PEGs protective effects.

## Supporting information

S1 Table XL spreadsheet of DESeq2output for 24-hour time point for sciatic nerve. (XLSX)

S2 Table XL spreadsheet of DESeq2output for 24-hour time point for tibialis muscle. (XLSX)

S3 Table XL spreadsheet of DESeq2output for 4-week time point for sciatic nerve. (XLSX)

S4 Table XL spreadsheet of DESeq2output for 4-week time point for tibialis muscle. (XLSX)

S5 Table Complete list of GO terms from Table 2. (DOCX)

S6 Table Complete list of GO terms from Table 5. (DOCX)
