# Supplementary material for: An analysis of differential gene expression in peripheral nerve and muscle utilizing RNA sequencing after polyethylene glycol nerve fusion in a rat sciatic nerve injury model
Source: PLoS One. 2024 Sep 4;19(9):e0304773. doi: 10.1371/journal.pone.0304773 (PMC11373823; doi:10.1371/journal.pone.0304773)
Supplement: S5 Table — (DOCX) [file pone.0304773.s005.docx]

**Supplementary Table 5**

**Table 2.** **All 97 pathways significantly affected by PEG in sciatic nerve at 4 weeks (GO Biological Process)**

| Term | P value | Corrected p value |
| --- | --- | --- |
| Single-Multicellular Organism Process | 2.92E-13 | 9.11E-10 |
| System development | 3.39E-13 | 9.11E-10 |
| Multicellular Organism Development | 5.47E-12 | 9.79E-09 |
| Single-Organism Developmental Process | 1.97E-10 | 2.52E-07 |
| Animal Organ Development | 2.35E-10 | 2.52E-07 |
| Developmental Process | 4.08E-10 | 3.65E-07 |
| Anatomical Structure Development | 4.83E-10 | 3.71E-07 |
| Single-Organism Process | 1.33E-09 | 8.92E-07 |
| Nervous System Development | 9.06E-08 | 5.40E-05 |
| Ion Transport | 2.61E-07 | 1.40E-04 |
| Response to Organic Cyclic Compound | 3.32E-07 | 1.62E-04 |
| Cellular Response to Organic Substance | 7.27E-07 | 3.17E-04 |
| Response to Organic Substance | 7.67E-07 | 3.17E-04 |
| Tissue Development | 9.45E-07 | 3.62E-04 |
| Peripheral Nervous System Development | 1.66E-06 | 5.57E-04 |
| Cell Differentiation | 1.66E-06 | 5.57E-04 |
| Lipid Metabolic Process | 2.56E-06 | 8.09E-04 |
| Response to Endogenous Stimulus | 4.47E-06 | 1.33E-03 |
| Ion Transmembrane Transport | 5.70E-06 | 1.61E-03 |
| Sodium Ion Transport | 1.06E-05 | 2.82E-03 |
| Sterol Biosynthetic Process | 1.10E-05 | 2.82E-03 |
| Inorganic Ion Transmembrane Transport | 1.54E-05 | 3.73E-03 |
| Metal Ion Transport | 1.60E-05 | 3.73E-03 |
| Cellular Response to Growth Factor Stimulus | 2.03E-05 | 4.49E-03 |
| Cation Transport | 2.09E-05 | 4.49E-03 |
| Cellular Developmental Process | 2.22E-05 | 4.49E-03 |
| Organic Hydroxy Compound Metabolic Process | 2.27E-05 | 4.49E-03 |
| Cell-Cell Signaling | 2.34E-05 | 4.49E-03 |
| Anion Transport | 2.46E-05 | 4.55E-03 |
| Cell Surface Receptor Signaling Pathway | 3.04E-05 | 5.43E-03 |
| Single-Organism Transport | 3.17E-05 | 5.49E-03 |
| Cellular Response to Chemical Stimulus | 3.98E-05 | 6.68E-03 |
| Multicellular Organismal Process | 4.89E-05 | 7.79E-03 |
| Response to External Stimulus | 5.05E-05 | 7.79E-03 |
| Regulation of Signaling | 5.08E-05 | 7.79E-03 |
| Single-ORganism Cellular Process | 5.58E-05 | 8.03E-03 |
| Neurogenesis | 5.60E-05 | 8.03E-03 |
| Enzyme Linked Receptor Protein Signaling Pathway | 5.81E-05 | 8.03E-03 |
| Transmembrane Transport | 5.83E-05 | 8.03E-03 |
| Response to Growth Factor | 6.48E-05 | 8.57E-03 |
| Cellular Response to Endogenous Stimulus | 6.54E-05 | 8.57E-03 |
| Steroid Biosynthetic Process | 7.76E-05 | 9.91E-03 |
| Regulation of Response to Stimulus | 8.25E-05 | 1.03E-02 |
| Regulation of Cell Communication | 8.81E-05 | 1.07E-02 |
| Single-Organism Localization | 9.12E-05 | 1.09E-02 |
| Signal Release | 9.53E-05 | 1.11E-02 |
| Steroid Metabolic Process | 9.92E-05 | 1.13E-02 |
| Neuron Projection Morphogenesis | 1.03E-04 | 1.15E-02 |
| Myelination | 1.06E-04 | 1.16E-02 |
| Sterol Metabolic Process | 1.16E-04 | 1.25E-02 |
| Ensheathment of Neurons | 1.27E-04 | 1.31E-02 |
| Axon Ensheathment | 1.27E-04 | 1.31E-02 |
| Localization | 1.31E-04 | 1.33E-02 |
| Cholesterol Biosynthetic Process | 1.42E-04 | 1.41E-02 |
| Regulation of Hormone Levels | 1.44E-04 | 1.41E-02 |
| Secondary Alcohol Biosynthetic Process | 1.68E-04 | 1.59E-02 |
| Regulation of Multicellular Organismal Process | 1.69E-04 | 1.59E-02 |
| Alcohol Biosynthetic Process | 1.72E-04 | 1.59E-02 |
| Regulation of Signal Transduction | 1.80E-04 | 1.64E-02 |
| Sodium Ion Transmembrane Transport | 1.93E-04 | 1.71E-02 |
| Sensory Perception of Mechanical Stimulus | 1.95E-04 | 1.71E-02 |
| Cell Development | 2.20E-04 | 1.90E-02 |
| Negative Regulation of Multicellular Organismal Process | 2.39E-04 | 2.03E-02 |
| Anatomical Structure Morphogenesis | 2.49E-04 | 2.08E-02 |
| Cell Morphogenesis Involved in Neuron Differentiation | 2.52E-04 | 2.08E-02 |
| Antigen Processing and Presentation of Peptide Antigen via MHC Class II | 2.56E-04 | 2.09E-02 |
| Neuron Differentiation | 2.81E-04 | 2.25E-02 |
| Axonogenesis | 3.11E-04 | 2.42E-02 |
| Sensory Perception of Sound | 3.12E-04 | 2.42E-02 |
| Secondary Alcohol Metabolic Process | 3.17E-04 | 2.43E-02 |
| Regulation of Cell Proliferation | 3.53E-04 | 2.65E-02 |
| Neuron Development | 3.56E-04 | 2.65E-02 |
| Response to Nutrient | 3.65E-04 | 2.69E-02 |
| Response to Extracellular Stimulus | 3.71E-04 | 2.69E-02 |
| Response to Lipid | 3.89E-04 | 2.79E-02 |
| Cation Transmembrane Transport | 4.04E-04 | 2.82E-02 |
| Muscle Organ Development | 4.09E-04 | 2.82E-02 |
| Response to Hormone | 4.09E-04 | 2.82E-02 |
| Intracellular Signal Transduction | 4.29E-04 | 2.92E-02 |
| Regulation of Biological Quality | 4.71E-04 | 3.16E-02 |
| Regulation of System Process | 4.94E-04 | 3.25E-02 |
| Chemical Homeostasis | 4.96E-04 | 3.25E-02 |
| Cellular Lipid Metabolic Process | 5.26E-04 | 3.40E-02 |
| Neuron Projection Development | 5.33E-04 | 3.41E-02 |
| Lipid Biosynthetic Process | 5.58E-04 | 3.53E-02 |
| Response to Ketone | 5.81E-04 | 3.62E-02 |
| Generation of Neurons | 5.92E-04 | 3.63E-02 |
| Organic Hydroxy Compound Biosynthetic Process | 5.97E-04 | 3.63E-02 |
| Small Molecule Biosynthetic Process | 6.03E-04 | 3.63E-02 |
| Secretion | 7.72E-04 | 4.58E-02 |
| Antigen Processing and Presentation of Peptide or Polysaccharide Antigen via MHC Class II | 7.76E-04 | 4.58E-02 |
| Hormone Transport | 7.91E-04 | 4.61E-02 |
| Inorganic Cation Transmembrane Transport | 8.36E-04 | 4.82E-02 |
| Cholesterol Metabolic Process | 8.48E-04 | 4.82E-02 |
| Transport | 8.53E-04 | 4.82E-02 |
| Positive Regulation of Cell Communication | 8.84E-04 | 4.94E-02 |
