# Supplementary material for: An analysis of differential gene expression in peripheral nerve and muscle utilizing RNA sequencing after polyethylene glycol nerve fusion in a rat sciatic nerve injury model
Source: PLoS One. 2024 Sep 4;19(9):e0304773. doi: 10.1371/journal.pone.0304773 (PMC11373823; doi:10.1371/journal.pone.0304773)
Supplement: S6 Table — (DOCX) [file pone.0304773.s006.docx]

**Supplementary Table 6**

**Table 5**. **All 52 pathways significantly affected by PEG in tibialis anterior muscle 24 hours (GO Biological Process).**

| Term | P value | Corrected p value |
| --- | --- | --- |
| Cell Adhesion | 4.61E-11 | 1.19E-07 |
| Response to Lipopolysaccharide | 5.49E-11 | 1.19E-07 |
| Inflammatory Response | 1.53E-10 | 2.21E-07 |
| Cytokine-Mediated Signaling Pathway | 3.64E-09 | 3.95E-06 |
| Integrin-Mediated Signaling Pathway | 4.65E-09 | 4.03E-06 |
| Angiogenesis | 2.73E-08 | 1.98E-05 |
| Neutrophil chemotaxis | 9.11E-08 | 5.65E-05 |
| Positive Regulation of T Cell Proliferation | 1.30E-07 | 6.44E-05 |
| Positive Regulation of Cell Migration | 1.34E-07 | 6.44E-05 |
| Platelet Aggregation | 1.49E-07 | 6.47E-05 |
| Oxygen Transport | 5.72E-07 | 2.26E-04 |
| Collagen Fibril Organization | 7.98E-07 | 2.89E-04 |
| Cellular Response to Platelet-Derived Growth Factor Stimulus | 9.73E-07 | 3.14E-04 |
| Receptor-Mediated Endocytosis | 1.01E-06 | 3.14E-04 |
| Positive Regulation of Neutrophil Chemotaxis | 1.72E-06 | 4.67E-04 |
| Leukocyte Cell-Cell Adhesion | 1.72E-06 | 4.67E-04 |
| Innate Immune Response | 2.46E-06 | 6.27E-04 |
| Extracellular Matrix Organization | 3.43E-06 | 8.27E-04 |
| Response to Cytokine | 4.77E-06 | 1.09E-03 |
| Cartilage Development | 5.41E-06 | 1.17E-03 |
| Regulation of the Force of Heart Contraction | 7.15E-06 | 1.48E-03 |
| Actin Filament Bundle Assembly | 8.17E-06 | 1.61E-03 |
| Negative Regulation of Apoptotic Process | 8.73E-06 | 1.65E-03 |
| Negative Regulation of Cell Proliferation | 1.07E-05 | 1.93E-03 |
| Positive Regulation of Angiogenesis | 1.91E-05 | 3.23E-03 |
| Complement Activation | 1.95E-05 | 3.23E-03 |
| Positive Regulation of Phagocytosis | 2.08E-05 | 3.23E-03 |
| Positive Regulation of Cell-Substrate Adhesion | 2.08E-05 | 3.23E-03 |
| Cell Migration | 3.01E-05 | 4.51E-03 |
| Negative Regulation of Blood Pressure | 3.35E-05 | 4.85E-03 |
| Response to Mechanical Stimulus | 3.65E-05 | 5.11E-03 |
| Response to Drug | 6.02E-05 | 8.17E-03 |
| Aging | 8.20E-05 | 1.08E-02 |
| Chronic Inflammatory Response | 8.60E-05 | 1.10E-02 |
| Negative Regulation of Inflammatory Response | 1.02E-04 | 1.26E-02 |
| Regulation of Cell Proliferation | 1.25E-04 | 1.51E-02 |
| Positive Regulation of Gene Expression | 1.64E-04 | 1.92E-02 |
| Lymph Node Development | 1.76E-04 | 1.98E-02 |
| Wound Healing | 1.77E-04 | 1.98E-02 |
| Positive Regulation of Mast Cell Degranulation | 2.24E-04 | 2.43E-02 |
| Blood Coagulation | 2.80E-04 | 2.91E-02 |
| Receptor Internalization | 2.81E-04 | 2.91E-02 |
| Endodermal Cell Differentiation | 3.00E-04 | 3.03E-02 |
| Response to Hypoxia | 3.52E-04 | 3.45E-02 |
| Positive Regulation of Apoptotic Process | 3.58E-04 | 3.45E-02 |
| Immunological Synapse Formation | 3.82E-04 | 3.60E-02 |
| Positive Regulation of GTPase Activity | 3.93E-04 | 3.63E-02 |
| Phagocytosis | 4.21E-04 | 3.81E-02 |
| Chondrocyte Development | 4.95E-04 | 4.38E-02 |
| Cellular Response to Interleukin-1 | 5.54E-04 | 4.72E-02 |
| Ossification | 5.54E-04 | 4.72E-02 |
| Cell Morphogenesis | 5.91E-04 | 4.93E-02 |
